# Sensors Integrated Control of PEMFC Gas Supply System Based on Large-Scale Deep Reinforcement Learning

**DOI:** 10.3390/s21020349

**Published:** 2021-01-06

**Authors:** Jiawen Li, Tao Yu

**Affiliations:** College of Electric Power, South China University of Technology, Guangzhou 510640, China; eplijiawen@mail.scut.edu.cn

**Keywords:** distributed deep reinforcement learning, edge-cloud collaborative multiple tricks distributed deep deterministic policy gradient, PEMFC, integrated control of gas supply system

## Abstract

In the proton exchange membrane fuel cell (PEMFC) system, the flow of air and hydrogen is the main factor influencing the output characteristics of PEMFC, and there is a coordination problem between their flow controls. Thus, the integrated controller of the PEMFC gas supply system based on distributed deep reinforcement learning (DDRL) is proposed to solve this problem, it combines the original airflow controller and hydrogen flow controller into one. Besides, edge-cloud collaborative multiple tricks distributed deep deterministic policy gradient (ECMTD-DDPG) algorithm is presented. In this algorithm, an edge exploration policy is adopted, suggesting that the edge explores including DDPG, soft actor-critic (SAC), and conventional control algorithm are employed to realize distributed exploration in the environment, and a classified experience replay mechanism is introduced to improve exploration efficiency. Moreover, various tricks are combined with the cloud centralized training policy to address the overestimation of Q-value in DDPG. Ultimately, a model-free integrated controller of the PEMFC gas supply system with better global searching ability and training efficiency is obtained. The simulation verifies that the controller enables the flows of air and hydrogen to respond more rapidly to the changing load.

## 1. Introduction

The accelerating growth of the global economy in recent years has been accompanied by increasing consumption of fossil fuels and attendant carbon emissions. The environmental pollution caused by traditional fossil fuel energy consumption (represented by oil, gas, and coal) poses an existential threat to humanity and ecosystems. In response, governments and scientists worldwide are directing more resources into renewable energy (‘green’) technology. Fuel cells that can directly convert chemical energy into electrical energy via chemical reactions are widely recognized as a valuable energy source. Among them, the proton exchange membrane fuel cell (PEMFC) has attracted more focus due to its fast start-up ability, high energy conversion rate, low working temperature, lightweight properties, and resistance to external natural conditions [1].

The PEMFC system generally includes four parts: gas supply system, thermal management system, water management system, and the energy control system. The gas supply system includes airflow control and hydrogen flow control. The airflow affects the oxygen excess ratio (OER) of the PEMFC. If the airflow is insufficient, oxygen starvation will occur, causing the output voltage of the PEMFC to decline sharply; if the airflow is excessive, oxygen saturation will occur, leading to an increase in the parasitic power, which affects the output characteristics of the PEMFC. Therefore, reasonable control of airflow is required so that the net power of the PEMFC remains stable [2,3,4]. Similarly, hydrogen flow has a significant impact on the output voltage of the PEMFC, in that reasonable hydrogen flow control enables the output voltage to respond timeously to load changes, thus stabilizing the output voltage and power. A large number of previous studies on PEMFC control have focused mainly on control of air flow and hydrogen flow. There are two general consensuses concerning these variables. Firstly, it is widely assumed that, provided that the hydrogen storage is sufficient and the flow control response is rapid, the PEMFC can accurately and rapidly satisfy the airflow demand; secondly, provided that there is a sufficient supply of air and its flow can be controlled rapidly, the PEMFC can meet the demand of the hydrogen flow control system timeously. However, with air or hydrogen supply systems it is often difficult to ensure sufficient gas storage and guarantee instantaneous response at any time because the relationship between gas flow rate and output voltage is non-linear and the response of the output voltage is delayed. Furthermore, the flow of the two gases jointly affects the output voltage and net power of the PEMFC. If the coordination between the two supply systems is insufficient, the output stability of the PEMFC will be seriously affected.

To solve the above problems, Woon et al. proposed a multi-input multi-output (MIMO) high order sliding mode algorithm to simultaneously control the air flow and hydrogen flow of the PEMFC. They considered the coordination between the control of the two gas streams, enabling the output voltage to remain in a stable state under different load conditions [5]. However, the high-frequency discontinuous switching items in the sliding mode controller make the actual level of control by the system discontinuous over time. As a result, the sliding mode controller will inevitably cause unfavorable chattering problems to affect the output voltage of the PEMFC. Therefore, this method cannot be applied to an actual PEMFC system. Sankar et al. put forward a sliding mode observer (SMO)-based nonlinear multivariable sliding mode controller (SMC) and globally linearizing controller (GLC) for a PEMFC; it employs a non-linear observer to improve the control performance of the algorithm and reduce chattering, thus realizing comprehensive optimal control of oxygen and hydrogen [6]. However, this method is complicated and to date has not proved to be a practical real-world solution. Wang et al. adopted a robust controller which regulates the flow of oxygen and hydrogen. Their PEMFC was modeled as a MIMO system, with air and hydrogen flow rates as the inputs and stack voltage and current as the outputs, which enables stable output voltage as well as lower hydrogen consumption [7,8]. Nevertheless, the steady-state accuracy of their PEMFC system proved to be weak since the robust controller generally does not work in the optimal state, and the underlying calculations are complicated. Thus, it is difficult for this method to be implemented in practice. On the basis of the designed state-space mathematical model, He et al. used a proportion integral (PI) controller and state feedback controller to control the flow rate of hydrogen and air respectively, so as to realize the coordinated control of the two gases [9]. However, their PI controllers and feedback controllers are incompatible with nonlinear systems (i.e., PEMFC), and their experimentation did not consider the control performance in achieving optimal control. Drawing on expert system and fuzzy control theory, Almeida et al. proposed a comprehensive intelligent controller that comprehensively considers the coordination of four systems: gas control, thermal management, water management, and energy control. However, the method cannot easily be applied to real-world systems due to the inefficient search ability and absence of self-learning in traditional expert systems [10]. Additionally, Almeida et al. proposed a method for realizing optimal control of PEMFC gas streams based on the parameterized cerebella model articulation controller (P-CMAC), whereby the voltage can be controlled by manipulating the flow rates of hydrogen and air [11]. This control strategy amounts to an approximate optimal control strategy; nevertheless, the neural network used in this method was a simple artificial neural network, resulting in extremely low robustness of the controller. In addition, the specific performance of this method was determined by the quality of the samples during offline training. Therefore, the method is lacking in self-correction ability, and it cannot ensure optimal control due to insufficient training.

Although the aforementioned algorithms enable coordinated control of the gas system, most of them do not employ a simple, high-performance control strategy, which would realize optimal control of the output voltage as well as appropriate oxygen excess rate. Thus, a simple model-free control algorithm is required so that a simpler, more practical method for controlling the gas supply system of the PEMFC can be realized, one which ensures coordination between hydrogen and oxygen supplies in the control process. The deep deterministic policy gradient (DDPG) algorithm in deep reinforcement learning is a model-free algorithm [12,13,14]. It combines the perception of deep learning with the decision-making ability of reinforcement learning and is characterized by strong adaptive ability, timely response, and accurate control. In contrast with conventional control methods, DDPG permits full interaction with the environment [15,16] and can be used to address the uncertainty in nonlinear control. Besides, controllers based on DDPG can realize MIMO control and are now being applied to various control fields [17,18,19]; to date, however, a working DDPG-based controller for a PEMFC has yet to be successfully demonstrated.

Furthermore, the DDPG algorithm still has many shortcomings, which make it an unsuitable candidate for the field of precision control. First of all, there is the problem of over-estimation of the Q value, causing the algorithm to fall into the local optimal solution, causing the final control strategy to deviate and yield poor control performance or even oscillation. Second, the DDPG algorithm is not sufficiently robust during offline training, and so the controller that directly uses this algorithm as a control algorithm has very low robustness. Third, the DDPG algorithm requires a long offline training period, which greatly limits its potential application in the industry.

In order to address these problems, several scholars have carried out a number of research studies. In response to the first problem, Fujimoto et al. proposed the twin delayed deep deterministic policy gradient (TD3) algorithm, whereby they introduced a policy delay update technique [20]. Their experimental results were moderately successful; however, they opined that solving Q value overestimation remains an intractable problem. Regarding the second problem, Horgan et al. proposed a new algorithm operating on a distributed reinforcement learning framework, which enables greater exploration ability [21]. Their results show that this algorithm did improve the algorithm’s exploration ability; however, it also learned more low-value samples, boosting offline training costs. Addressing the third problem, Schau et al. proposed a prior experience replay technique [22], which can sort samples according to their value and preferentially select samples with high value for training. Nevertheless, to date, the algorithm which only employs this technique remains ineffective. Another technique is the edge-cloud collaborative framework, a form of collaborative computing that has recently caught the imagination of the academic community. This framework can be used as a hardware architecture for deep reinforcement learning to obtain better computing power and to increase the exploration ability and optimization speed of the algorithm. A distributed reinforcement learning framework can be embedded in such an architecture and used to obtain better training results [23].

In view of the fact that DDPG suffers from the problem of Q value overestimation as well as the problem of low exploration ability (ascribed to its single exploration ability, in that it employs only one actor network to explore the environment), a new algorithm which can solve these problems and achieve better coordination control of air and hydrogen flow is proposed. A number of improvements to the original DDPG algorithm are presented. In this paper, an integrated gas supply system controller for a PEMFC based on the ECMTD-DDPG algorithm is presented.

This paper makes the following novel contributions to the field:

1. An integrated controller based on deep reinforcement learning is proposed. This is a combined controller that integrates the flow controllers for air and hydrogen. It can formulate the control strategy according to different PEMFC states and output the motor voltage of the air compressor and hydrogen flow simultaneously. Hence, the real-time control requirements of the PEMFC under different operating conditions can be met.

2. An ECMTD-DDPG algorithm is proposed for the aforementioned framework. This is a distributed deep reinforcement learning algorithm with the edge-cloud collaborative framework. It is designed with multiple edge explorers and a cloud learner. Second, the edge exploration policy is adopted in edge explorers in order to increase exploration efficiency. Third, the classified experience replay mechanism is introduced in order to improve training efficiency. Fourth, the cloud learner employs clipping multi-Q learning, delay policy updating, and smooth regularization of target policy to solve overestimation of Q value in DDPG. Finally, a control algorithm with better robustness and adaptability is proposed. The simulation results below demonstrate that the proposed method can effectively control the output voltage of the PEMFC and ensure stable operation of the system.

## 2. The Principle of the Gas Supply System

**Figure 1:**
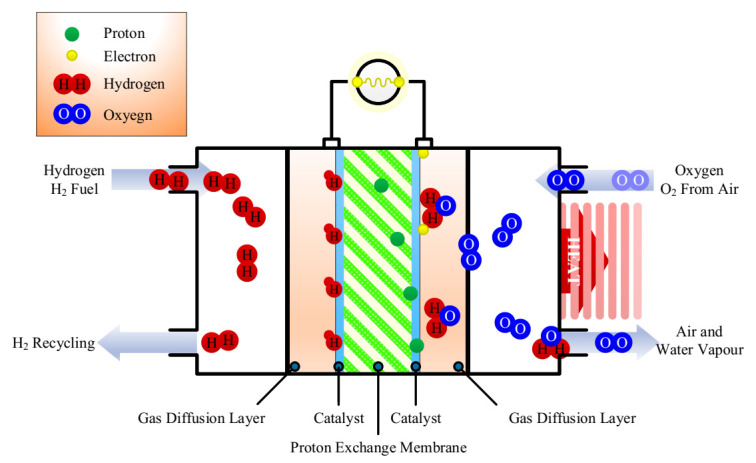
Chemical reaction in proton exchange membrane fuel cell (PEMFC).

The chemical reactions are shown in Figure 1. First, hydrogen enters the PEMFC polar plate flow channel from the anode inlet. (The polar plate flow channel is attached to the anode gas diffusion layer.) Then, the hydrogen passes through the gas diffusion layer to the catalytic layer and undergoes a reduction reaction under the action of the catalyst, separating into hydrogen ions and electrons. The proton exchange membrane has selective permeability, which allows hydrogen ions to pass through whilst blocking the electrons. Therefore, hydrogen ions can pass through the proton exchange membrane to reach the cathode, while electrons can only reach the cathode through the external circuit. The flow of electrons in the external circuit constitutes direct current and supplies power to the load. At the cathode, the hydrogen ions passing through the proton exchange membrane and the electrons arriving through the external circuit merge, before arriving at the cathode entrance. The oxygen passing through the cathode gas diffusion layer to the catalytic layer is combined with hydrogen, and the oxidation reaction occurs under the action of the catalyst to generate water and heat, which are then removed [1,2].

### 2.1. The Dynamic Control Model of the Voltage

In the PEMFC, the pressure of hydrogen is subject to the influence of the inlet flux of hydrogen, the outlet flux of hydrogen, and the flux of hydrogen being consumed by the chemical reaction. According to the principle of conservation of matter, the ideal gas state can be expressed as follows:

(1) $$\frac{V_{a}}{RT}\frac{dP_{H_{2}}}{dt}=m_{H_{2},i}-K_{a}\left(P_{H_{2}}-P_{H_{2},B}\right)-\frac{0.5Ni}{F}$$

where *V*_a_ is total volume of flow field in the anode, *R* is the gas constant, *T* is the working temperature, *P*_H2_ is the partial pressure of hydrogen, *m*_H2,i_ is the inflow flux of hydrogen, *K*_a_ is the flux coefficient in the anode, *P*_H2,B_ is the hydrogen removal pressure, *N* is the number of single fuel cells, *F* is the Faraday constant, and *i* is the load current.

Likewise, the oxygen pressure is influenced by the inlet flux of air, the outlet flux of air and the flux of air consumed by the chemical reaction. According to the continuity principle, the masses of the two kinds of components in the cathode, oxygen and nitrogen, are balanced. Based on the conservation of matter, the continuity equation for oxygen and nitrogen can be expressed as follows:

(2) $$\frac{dm_{O_{2}}}{dt}=W_{O_{2},in}-W_{O_{2},\;out\;}-W_{O_{2},\;ret\;}$$

(3) $$\frac{dm_{N_{2}}}{dt}=W_{N_{2},in}-W_{N_{2},\;out\;}$$

The OER is shown as follows:

(4) $$\lambda_{O_{2}}=\frac{W_{O_{2},in}}{W_{O_{2},ret}}$$

where $m_{O_{2}}$ and $m_{N_{2}}$ are the masses of oxygen and nitrogen respectively; $W_{O_{2,in}}$ and $W_{N_{2,in}}$ represent the flows of oxygen and nitrogen entering the stack; $W_{O_{2,out}}$ and $W_{N_{2,out}}$ are the flows of oxygen and nitrogen flowing out of stack; and, $W_{O_{2,ret}}$ is the flow of oxygen consumed due to reaction in the stack.

The oxygen pressure characteristic equation can be obtained as follows:

(5) $$\frac{V_{c}}{RT}\frac{dP_{O_{2}}}{dt}=m_{O_{2},i}-K_{c}\left(P_{O_{2}}-P_{O_{2},B}\right)-\frac{0.2Ni}{F}$$

where *V*_c_ is the total volume of flow field in the anode, $P_{O_{2}}$ is the partial pressure of hydrogen, *K*_c_ is the flux coefficient in the anode, and $P_{O_{2},B}$ is the hydrogen removal pressure.

The thermodynamic electromotive force is expressed as follows:

(6) $$E_{\mathrm{Nernst}\;}=1.229-0.00085(T-298.15)+0.000043T\left(\ln P_{H_{2}}+\frac{1}{2}\ln P_{O_{2}}\right)$$

where *E*_Nernst_ is the thermodynamic electromotive force. The ohmic overpotential is as follows:

(7) $$V_{\mathrm{ohmic}\;}=-i\left(R_{M}+R_{c}\right)$$

where *V*_ohmic_ is the ohmic overpotential, *R*_M_ is the equivalent membrane impedance of the proton membrane, and *R* is the impedance that prevents the proton from passing through the proton membrane. There exists an electrical double layer in the PEMFC [1,2]. If equivalent capacitors *C* are connected to both ends of the polarization resistor in parallel, the PEMFC will exhibit excellent dynamic properties. The differential equation of a single fuel cell is as follows:

(8) $$\frac{dv_{d}}{dt}=\frac{i}{C}-\frac{v_{d}}{q}$$

where *v*_d_ is the total polarization overvoltage.

Having taken into account the thermodynamic properties, mass transfer and dynamic properties, the output voltage of the PEMFC can be expressed as follows:

(9) $$V_{\mathrm{cell}\;}=E_{\mathrm{Nernst}\;}-V_{\mathrm{ohmic}\;}-v_{d}$$

### 2.2. Supply Pipe

Taking into account the principles of conservation of mass and conservation of energy, the supply pipe in the cathode can be represented by the following equations:

(10) $$\frac{dm_{\mathrm{sm}}}{dt}=W_{\mathrm{cp}}-W_{\mathrm{sm}}$$

(11) $$\frac{dp_{\mathrm{sm}}}{dt}=\frac{\gamma R_{a}}{V_{\mathrm{sm}}}\left(W_{\mathrm{cp}}T_{\mathrm{cp}}-W_{\mathrm{sm}}T_{\mathrm{sm}}\right)$$

where *m*_sm_ is the mass of air in the supply pipe, *p*_sm_ is the pressure in the supply pipe, *γ* is the heat ratio coefficient of air, *R_a_* is the gas constant of air, *V*_sm_ is the supply pipe volume, *T*_cp_ is the temperature at which the compressor presses in air, *T*_sm_ is the temperature of the air in the supply pipe, *W*_cp_ is the flux through the compressor, and *W*_sm_ is the mass flux through the supply pipe.

For the supply pipe, the flow rate of the gas flowing into the pipe is equal to the flow rate of the air compressor ${\;W}_{cp}$, and the flow rate of the gas flowing out is ${\;W}_{sm,\;out\;}$. Since the pressure difference between the supply pipe and the cathode is relatively small, the flow rate of the gas flowing out is expressed as follows:

(12) $$W_{sm,\mathrm{out}}=k_{sm,\mathrm{out}}\left(p_{\mathrm{sm}}-p_{\mathrm{ca}}\right)$$

where $k_{sm,out}$ is the outlet flow constant of the supply pipe, and *P*_ca_ is the cathode gas pressure.

### 2.3. Return Pipe

The change of temperature needs to be considered in the design of the return pipe. The gas temperature *T_m_* in the return pipe is equal to the temperature of the gas leaving the cathode. Pressure *p*_rm_ of return pipe can be obtained based on the *l* conservation of mass and the ideal gas law, i.e.,

(13) $$\frac{dp_{\mathrm{rm}}}{dt}=\frac{R_{a}T_{\mathrm{rm}}}{V_{m}}\left(W_{\mathrm{ca}}-W_{\mathrm{rm}}\right)$$

where *W*_ca_ is the air flux in the stack cathode, *W*_rm_ is the air flux at the outlet of the return pipe, and *V*_rm_ is the volume of the return pipe.

Since the pressure drop between the return manifold and the atmospheric is relatively large, the equations of return manifold exit flow are as follows:

(14) $$W_{rm,out}=\frac{C_{D,rm}A_{T,rm}p_{rm}}{\sqrt{\bar{R}T_{rm}}}{\left(\frac{p_{atm}}{p_{rm}}\right)}^{\frac{1}{\gamma }}{\left\{\frac{2\gamma }{\gamma -1}\left[1-{\left(\frac{p_{atm}}{p_{rm}}\right)}^{\frac{\gamma -1}{\gamma }}\right]\right\}}^{\frac{1}{2}}\;\;\mathrm{for}\;\;\frac{p_{atm}}{p_{rm}}>{\left(\frac{2}{\gamma +1}\right)}^{\frac{\gamma }{\gamma -1}}$$

and

(15) $$W_{rm,out}=\frac{C_{D,rm}A_{T,rm}p_{rm}}{\sqrt{RT_{rm}}}\gamma^{\frac{1}{2}}{\left(\frac{2}{\gamma +1}\right)}^{\frac{\gamma +1}{2(\gamma -1)}}\;\;\mathrm{for}\;\;\frac{p_{atm}}{p_{rm}}\leq {\left(\frac{2}{\gamma +1}\right)}^{\frac{\gamma }{\gamma -1}}$$

where $A_{T,rm}$ is the return manifold throttle area, $C_{D,rm}$ is the return manifold throttle discharge coefficient, *p_rm_* is the return manifold pressure, and *T_sm_* is the return manifold temperature, *p_atm_* is atmospheric pressure.

### 2.4. Air Compressor

The dynamic properties of the compressor can be described using a rotation model [1], i.e.,

(16) $$\left\{\begin{array}{l} J_{\mathrm{cp}}\frac{d\omega_{\mathrm{cp}}}{dt}=\tau_{\mathrm{cm}}-\tau_{\mathrm{cp}}\\ \tau_{\mathrm{cm}}=\eta_{\mathrm{cm}}\frac{k_{t}}{R_{\mathrm{cm}}}\left(v_{\mathrm{cm}}-k_{v}\omega \right)\\ \tau_{\mathrm{cp}}=W_{\mathrm{cp}}\frac{C_{p}T_{\mathrm{atm}}}{\omega \eta_{\mathrm{cp}}}\left[{\left(\frac{p_{\mathrm{sm}}}{p_{\mathrm{atm}}}\right)}^{\frac{\gamma -1}{\gamma }}-1\right] \end{array}\right.$$

where *J*_cp_ is the rotational inertia of the compressor; *ω*_cp_ is the speed of the compressor; *τ*_cm_ is the motor torque of the compressor; *τ*_cp_ is the load torque of the compressor; *k*_t_, *R*_cm_, and *k*_v_ are the motor constants; *η*_cm_ is the mechanical efficiency of the motor; *v*_cm_ is the control voltage of the motor; *C*_p_ is the specific heat capacity of air; *η*_cp_ represents the efficiency of the compressor; and *p*_atm_ and *T*_atm_ represent the atmospheric pressure and temperature, respectively.

### 2.5. The Control Principle of Gas Supply System

**Figure 2:**
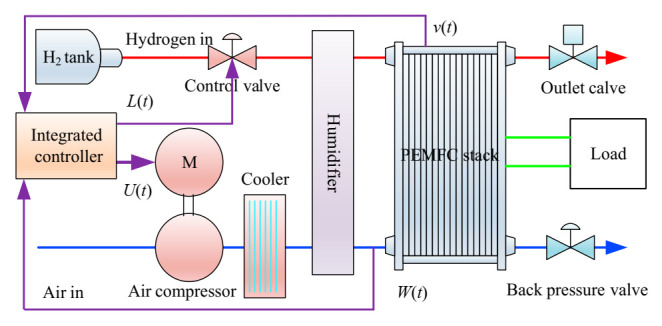
The control framework of integrated controller.

Research has indicated that there is an important correlation between the output voltage of the system and the flux of reactant gas. Since the output voltage of PEMFC is mainly affected by hydrogen flux and air flux, during modeling, both air flux and hydrogen flux are controlled by an intelligent controller based on EILMMA-DDPG. In this paper, a dynamic model of PEMFC is set up. The control objective is to keep output voltage and OER of PEMFC to stabilize at the optimal value v_st_* and *W_O_*_2_* by controlling the voltage of the motor for air compressor and the hydrogen flux. The control framework is shown in Figure 2.

## 3. ECMTD-DDPG Framework

### 3.1. Deep Reinforcement Learning

Deep reinforcement learning (DRL) is a kind of machine learning that is not model-based. This method aims at maximizing the accumulation of long-term rewards and allows agents to continuously learn the optimal actions in different states. In deep reinforcement learning, there are two types of commonly used algorithms. One type is value-based DRL, which includes deep Q learning (DQN) and deep double Q learning (DDQN). This type of algorithm is generally used to deal with problems with discrete action spaces. The advantage of value-based DRL is that convergence is fast and it does not fall easily into a local optimal solution. The other is policy-based DRL, which includes DDPG and TD3, etc. Policy-based DRL is often used to deal with problems with continuous action space, and is therefore a commonly used algorithm; however, this algorithm can fall easily into a local optimal solution.

In recent years, deep reinforcement learning algorithms have continued to develop, and distributed deep reinforcement learning using computer parallel computing capabilities have been proposed. The chief characteristic of distributed reinforcement learning is to use each agent in the system as the main body of learning. These agents learn the response policy to the environment and the mutual cooperation policy. In addition, they use the CPU or GPU of multiple computers to realize better computing processing power, so as to improve the performance of the algorithm. Moreover, they usually adopt the model of centralized training and decentralized execution. All agents in the algorithm correspond to a leader for centralized training such as the APEX-DDPG algorithm [22].

Edge cloud collaboration is a form of collaborative computing and is a relatively mature technology model.

An edge-oriented edge cloud collaboration approach is used in this paper, in that the Cloud is only responsible for initial training work, and the model is downloaded to the edge after the training is completed. Computing tasks are performed at the edge. The edge cloud collaboration framework is introduced in distributed deep reinforcement learning. In the proposed ECMTD-DDPG framework, the leader in distributed reinforcement learning is the cloud. It contains a variety of neural networks and is responsible for the initial centralized training model. The edge is the edge explorer, and each edge explorer contains a neural network. These networks are responsible for collecting data to assist the cloud for training while performing their own computing tasks in the online application process. During training, the role of DRL is to use a large amount of computer computing power at the edge to continuously search for optimization and exploration, and to deliver the explored information to the cloud for centralized training. Following the initial training, the DRL agent can directly make decisions through the trained model. Through the combination of such technologies, the integration of distributed reinforcement learning and edge-cloud collaboration technology is realized. Related common algorithms and edge-cloud collaboration frameworks are described below.

### 3.2. Common Policy Gradient Algorithms

#### 3.2.1. DDPG

In [14], a DDPG algorithm is proposed, which is a deterministic policy algorithm. DDPG employs two deep neural networks, namely, policy network and value function network. They correspond to policy function *π_ϕ_^j^*(*s*) and value function $Q_{\theta }\left(s,a\right)$ respectively, with their parameters of *ϕ* and *θ*. DDPG is designed to find an optimal policy *π_ϕ_* which maximizes the expected return value $J(\phi {)\;=\;E}_{s_{i}{\sim p}_{\pi }{,a}_{i}\sim \pi }[R_{0}]$. It employs the loss function to update the critics as Formula (17) and employs the policy gradient to update the actor policy as Formula (18)

(17) $$L=\frac{1}{N}\sum_{i}{\left(y_{i}-Q\left(s_{i},a_{i}|\theta^{Q}\right)\right)}^{2}$$

(18) $${\left.\nabla_{\theta^{\mu }}J\approx \frac{1}{N}\sum_{i}\nabla_{a}Q\left(s,a|\theta^{Q}\right)\right|}_{s=s_{i},a=\mu \left(s_{i}\right)}{\left.\nabla_{\theta^{\mu }}\mu \left(s|\theta^{\mu }\right)\right|}_{s_{i}}$$

where the ${\mu (s|\theta }^{\mu })$ is the policy which has the parameter of $\theta^{\mu }$, *L* is the loss function, *y_i_* is the target values.

#### 3.2.2. SAC

SAC, which is developed based on DDPG, is a stochastic policy gradient algorithm. Its typical feature is that entropy regularization is introduced to improve the randomness of action selection in the training process. For a policy of SAC, the greater the entropy, the higher the randomness of action selection. The expression of entropy is as follows:

(19) $$H\left(\pi \left(\cdot |s_{t}\right)\right)=-\sum_{t=1}^{\infty }\pi \left(\cdot |s_{t}\right)\log\pi \left(\cdot |s_{t}\right)$$

Then, the expression of optimal policy of SAC algorithm is as follows:

(20) $$\pi^{*}=\arg\max_{\pi }E_{\tau \sim \pi }\left[\sum_{t=0}^{\infty }\gamma^{t}\left(R\left(s_{t},a_{t},s_{t+1}\right)+\alpha H\left(\pi \left(\cdot |s_{t}\right)\right)\right)\right]$$

SAC algorithm adopts five neural networks, namely, policy network, two *Q* networks, and two *V* networks. Among them, the policy network outputs actions, the state value network *V*(*s*) outputs the value of current state, the target state value network outputs the value of next state, and two action value networks $Q_{1}\left(s,a\right)$,$\;Q_{2}\left(s,a\right)$ output the value of action selection.

### 3.3. ECMTD-DDPG

#### 3.3.1. Clipped Multi-Q Learning

Inspired by double deep Q-learning (DDQN) [12], ECMTD-DDPG employs the current actor network to select the optimal action and uses the target critic network to evaluate the policy. This process is encapsulated by Formula (21):

(21) $$y_{t}=r\left(s_{t},a_{t}\right)+\gamma Q_{\theta^{\prime }}\left(s_{t+1},\pi_{\phi }\left(s_{t+1}\right)\right)$$

In DDPG, the target actor network and the target critic network adopt the soft update mechanism [14], which establishes similarity between the current network and the target network. This, however, makes it hard to effectively separate the action selection from the policy evaluation. Therefore, the clipped double Q-learning method is adopted to calculate the target value, as expressed in Formula (22):

(22) $$y_{t}^{1}=r\left(s_{t},a_{t}\right)+\gamma \;\min_{i=1,2}Q_{\theta_{i}^{\prime }}\left(s_{t+1},\pi_{\phi_{1}}\left(s_{t+1}\right)\right)$$

The Cloud leader in ECMTD-DDPG employs an independent actor network and three critic networks. The actor network $\pi_{\phi_{1}}$ is updated according to the first critic network, and the target values *y_t_*^2^ and *y_t_*^3^ of other critic networks are equal to *y_t_*^1^.

#### 3.3.2. Policy Delayed Updating

The Cloud leader of ECMTD-DDPG updates the actor network after the critic network is updated for *d* times, so as to ensure that the actor network can be updated with low Q value error and thus improve the updating efficiency of the actor network.

#### 3.3.3. Smooth Regularization of Target Policy

ECMTD-DDPG introduces a regularization method to reduce the variance of the target, as expressed by Formula (23):

(23) $$y_{t}=r\left(s_{t},a_{t}\right)+E_{\varepsilon }\left[Q_{\theta^{\prime }}\left(s_{t+1},\pi_{\phi^{\prime }}\left(s_{t+1}\right)+\varepsilon \right)\right]$$

At the same time, by adding a random noise to the target policy and averaging on mini-batch, smooth regularization is realized:

(24) $$y_{t}=r\left(s_{t},a_{t}\right)+\gamma \min_{i=1,2}Q_{\theta_{t}}\left(s_{t+1},\pi_{\phi^{\prime }}\left(s_{t+1}\right)+\varepsilon \right)$$

(25) ε~clip(N(0,σ),−c,c)

#### 3.3.4. Distributed Training Method Based on Edge-Cloud Collaborative Framework

(1) Edge computing and cloud computing

Cloud computing is a centralized service, which has a large number of computing resources. All data is transmitted to the cloud computing center for processing, but it is difficult to ensure the timeliness and computing speed because of mass data.

Edge computing is a distributed computing architecture, which decomposes large-scale services that are originally handled by central nodes and distribute them to edge nodes. The purpose is to fully mobilize the computing power of network terminals, deal with computing problems more quickly and accurately in real time and reduce the cloud data communication.

Edge computing and cloud computing supplement each other. Specifically, cloud computing is a unified leader responsible for big data analysis of long-term data, while edge computing focuses on real-time processing and execution.

(2) Computing architecture

By referring to the above computing architecture, ECMTD-DDPG algorithm adopts a distributed reinforcement learning and training framework based on edge-cloud collaboration. There are two roles in the algorithm. The first role is the edge explorer based on the multi-exploration principle, which corresponds to the edge computing terminal in the edge-cloud collaborative framework. It acts to realize distributed exploration of the environment, so as to obtain more abundant training samples. The edge explorer includes 24 DDPG-edge explorers, 8 SAC-edge explorers, and 8 control algorithm edge explorers (CA-edge explorers) corresponding to different CPUs. They have fast computing speed and can realize real-time sampling.

Another role in the algorithm is a cloud leader, which includes two public experience pools, among which the cloud leader includes three critics and one actor, corresponding to one GPU. The cloud leader deals with a large amount of data in the public experience pools by collecting mini-batch and obtains the optimal control strategy by training. The parameter updating is slow without updating the neural network in real time.

In this paper, DDPG-edge explorer architecture includes only one actor network, and each has its own network model as well as environment. Different DDPG-edge explorer adopts different exploration principles, including greedy strategy, Gaussian noise, and Ornstein–Uhlenbeck (OU) noise. In different DDPG-edge explorers, the actor network adopts different exploration policies. The exploration policy of actor network in 8 edge-explorers is set as greedy strategy, which is named ε-DDPG-edge-explorers, that is, any action is selected in the action space with a certain probability.

(26) $$a_{\varepsilon }^{l}=\left\{\begin{array}{ll} \pi_{\theta }^{l}(s) & \mathrm{With}\;\varepsilon \;\mathrm{probability}\\ a_{\mathrm{rand}}^{l}{}_{\;} & \mathrm{With}\;1-\varepsilon \;\mathrm{probability}\; \end{array}\right.$$

The optimization policy of the actor network in 8 DDPG-edge explorers is set as OU noise, which is named OU-DDPG -edge-explorer. Different OU-DDPG-edge-explorers adopt random OU noise with different variance.

(27) $$a_{OU}^{j}=\pi_{\theta }^{j}(s)+N_{OU}^{j}$$

The optimization policy of the actor network in 8 DDPG-edge explorers is set as Gaussian noise, which is named Gaussian-DDPG-edge explorer. Different Gaussian-DDPG-edge explorers adopt random Gaussian noise with different variance.

(28) $$a_{\mathrm{Gaussian}}^{m}=\pi_{\theta }^{m}(s)+N_{Gaussian}^{m}$$

By adopting the exploration schemes based on the above different principles, the randomness and diversity of the explored samples can be increased.

SAC-edge explorer contains a complete SAC agent architecture, different network models, and environments, as described in Section 3.2.2. SAC-edge explorer adopts its own policy for exploration in different environments and puts the samples obtained from exploration into its own experience pool and the public experience pools in cloud leader. In addition, SAC-edge explorer regularly draws samples from its own experience pool for training and updates its own parameters according to the Formula (18).

CA-explorer has a control algorithm with different principles. By interacting with different environments, it can generate corresponding demonstration samples and guide cloud leader to learn.

A variety of edge explorers explore the environment in parallel. First, DDPG-edge explorer and SAC-edge explorer generates training samples *e_i_^DDPG^* = (*s_t_^i-DDPG^*, *a_t_^i-DDPG^*, *r_t_^i-DDPG^*, *s_t+_*_1_*^i-DDPG^*) and *e_i_^SAC^* = (*s_t_^i-SAC^*, *a_t_^i-SAC^*, *r_t_^i-SAC^*, *s_t+_*_1_*^i-SAC^*) based on their own environments, and CA-explorer generates the demonstration sample *e_i_^CA^* = (*s_t_^i-CA^*, *a_t_^i-CA^*, *r_t_^i-CA^*, *s_t+_*_1_*^i-CA^*) based on the environment. The conversion experience is added to two public experience pools according to the standard. Then, cloud leader draws mini-batch from the public experience pool according to the classification experience replay mechanism and keeps learning. Finally, the actor network in DDPG-edge explorer regularly updates its network parameters according to the latest network of actor from cloud leader.

#### 3.3.5. Guided Exploration Policy-Based Imitation Learning

In imitation learning, agents utilize human expert demonstration samples to improve their training efficiency. In order to increase the effect of human experts in the demonstration, the imitation learning proposed in this paper is to imitate the control strategy of which the parameters have been artificially regulated and considered to have optimized collaboration. The control output and data received by the controller in CA-edge explorer will be converted to the demonstration sample *e_i_^CA^* = (*s_t_^i-CA^*, *a_t_^i-CA^*, *r_t_^i-CA^*, *s_t+_*_1_*^i-CA^*) which is stored in the public experience pool.

**Figure 3:**
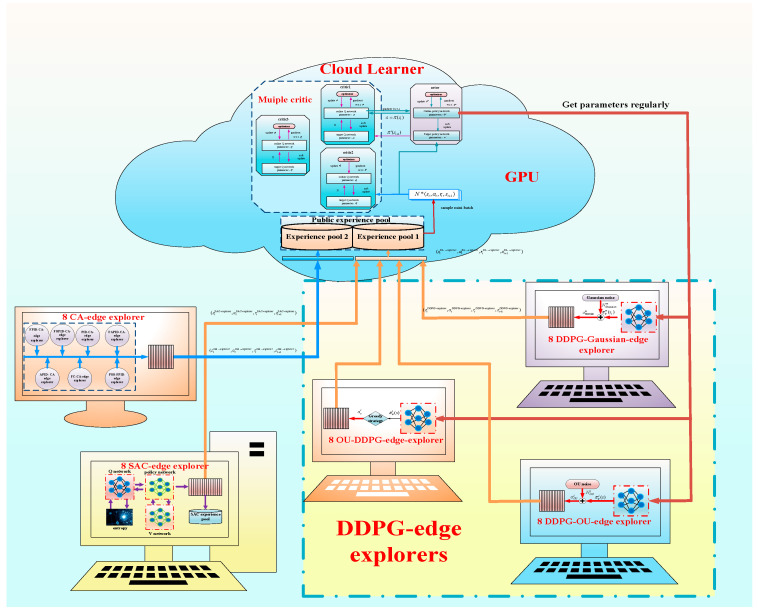
Distributed training framework of ECMTD-DDPG.

**Figure 4:**
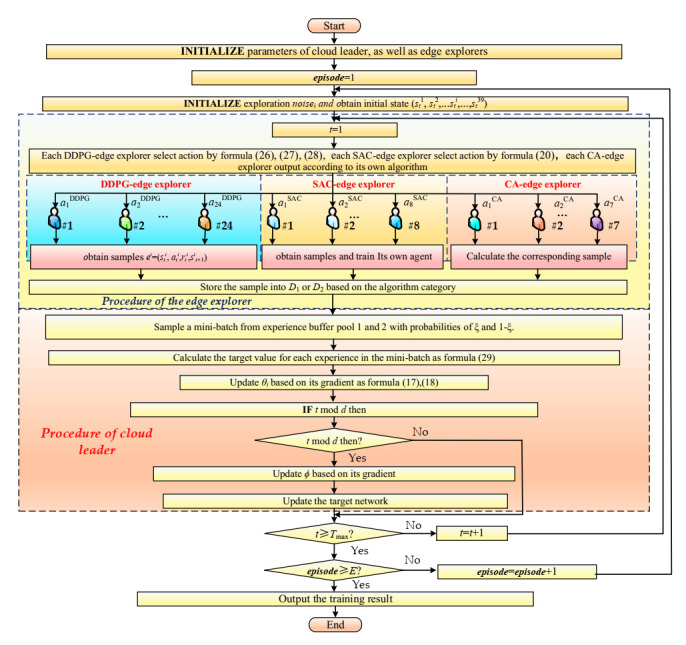
ECMTD-DDPG algorithm flow chart.

As shown in Figure 3, different CA-edge explorers adopt different control algorithms, including PID, fuzzy PID, PSO-optimized fuzzy PID (artificially setting air flow controller parameters and PSO-optimized hydrogen flow control parameters), fractional-order PID (FOPID), adaptive PID, fuzzy adaptive PID and fuzzy control, which are respectively named PID-CA-explorer, FPID-CA-explorer, PSO-FPID-CA-explorer, FOPID-CA-explorer, APID-CA-explorer, FAPID-CA-explorer, and FC-CA-explorer. These CA-explorers only collect samples and do not need to receive any information from the cloud leader. They provide diversified learning samples for the cloud leader. The specific algorithm flow is shown in Figure 4.

#### 3.3.6. Classified Experience Replay

In cloud leader, two independent public experience pools are employed for storing samples. When the network model is initialized, all samples in these two public experience pools are cleared. During training, samples collected by DDPG-edge explorer and SAC-edge explorer are put into experience pool 1, and samples collected by CA-edge-explorer are stored in experience pool 2. In the pre-learning, with respect to pool 1, the probability of getting *n_ξ_* samples is *ξ*. In pool 2, the probability of obtaining *n*_(1*-ξ*)_ samples is (1-*ξ*).

In order to enable agents to get more demonstration samples generated by CA-explorer in the early stage of training and learn more samples collected by DDPG-edge explorer as well as SAC-edge explorer in the later stage, the probability increases gradually with the increase of episodes, as shown in Formula (29).

(29) $$\xi =\left\{\begin{array}{ll} 0.7 & \mathrm{episodes}<1000\\ 0.8 & 1000<\mathrm{episodes}<2000\\ 0.9 & 2000<\mathrm{episodes}<3000\\ 1 & \mathrm{episodes}>3000 \end{array}\right.$$

## 4. Design of ECMTD-DDPG Integrated Controller

**Figure 5:**
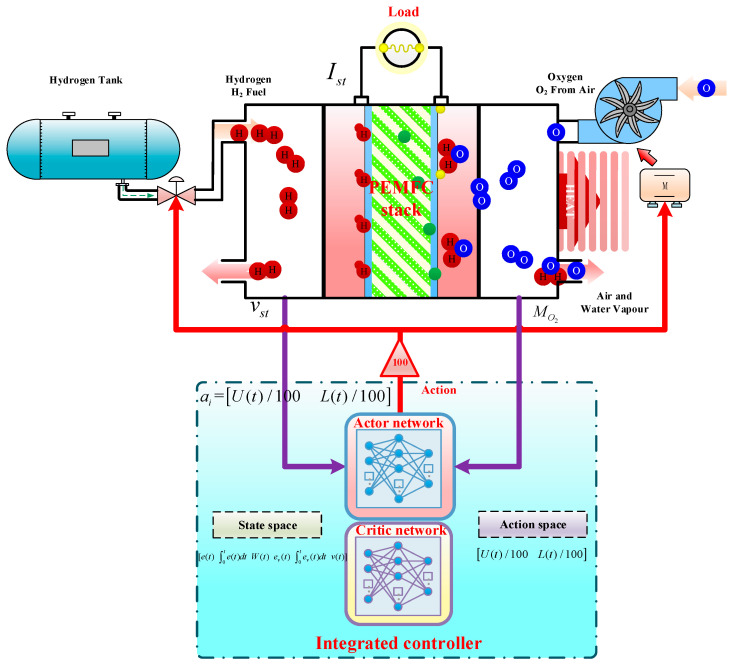
Integrated control of PEMFC gas supply system based on ECMTD-DDPG algorithm.

The integrated controller of the gas supply system described in this paper mainly has two control objectives: (1) to ensure proper OER or air flux; (2) to ensure stable output voltage under the condition of proper OER. The integrated controller indirectly controls the OER and output voltage of PEMFC by simultaneously controlling the motor voltage of air compressor and the flow of hydrogen according to the control error of air flow and output voltage in the system as well as the related states. The control interval is 0.01 s. The specific control framework, state space, and action space are shown in Figure 5.

### 4.1. Action Space

The action space is set as shown in Formula (30).

(30) $$\left\{\begin{array}{l} a=\left[U(t)/100\;L(t)/100\right]\\ 0\leq U(t)\leq U_{\max}\\ 0\leq L(t)\leq L_{\max} \end{array}\right.$$

where the *t* is the discrete time, *U*(*t*) is the motor voltage of air compressor; *U*_max_ is the upper limit of the motor voltage of the air compressor; *L*(*t*) is the hydrogen flow; and *L*_max_ is the upper limit of hydrogen flow.

### 4.2. State Space

State refers to the control error between air flow and ideal air flow of the input controller (*e*_o2_(*t*)), (*W*(*t*)), (*e*_v_(*t*)), its integral to *t* and output voltage *v*(*t*), state space is shown in Formula (31).

(31) $$\left[e_{O_{2}}(t)\;\;\int_{0}^{t}e_{O_{2}}dt\;\;W(t)\;\;e_{v}(t)\;\;\int_{0}^{t}e_{v}dt\;\;v(t)\right]$$

where the $e_{O_{2}}\left(t\right)$ refers to the control error between air flow and ideal air flow of the input controller, $\int_{0}^{t}e_{O_{2}}\left(t\right)$ refer to the $e_{O_{2}}\left(t\right)$ integral to *t*, *W*(*t*) refer to the air flow. *e_v_*(*t*) refer to the control error between output voltage and output voltage reference value of input controller.$\;\int_{0}^{t}e_{v}\left(t\right)$ refer to the $e_{v}(t)\;$ integral to *t*, *v*(*t*) refers to the output voltage of PEMFC.

### 4.3. Selection of Reward Function

The reward function is expressed as follows:

(32) $$r(t)=-\left[\mu_{1}e_{O_{2}}^{2}(t)+\mu_{2}e_{v}^{2}(t)\right]\;+\beta$$

(33) $$\beta =\left\{\begin{array}{ll} 0 & e_{O_{2}}^{2}(t)>0.01\;\mathrm{and}\;e_{v}^{2}(t)>0.09\\ 2.2 & e_{O_{2}}^{2}(t)\leq 0.01\;\mathrm{and}\;e_{v}^{2}(t)\leq 0.09\;\\ 1.1 & otherwise \end{array}\right.$$

where the *β* means the control reward item. According to the state of *e*_o2_(*t*) and *e*_v_(*t*), agents are given a proper positive reward.

## 5. Simulation

The parameters of the PEMFC in the simulation are from the reference [24] which is based on the real experimental data of FORD P2000 vehicle with 75 kW PEMFC [25,26,27]. The working temperature of PEMFC is considered to be approximately constant which is 353 K. In addition, the gases in the PEMFC are fully humidified. The simulation software package used is MATALB/Simulink version 9.9.0 (R2020b). The pre-learning parameters are shown in Table 1.

### 5.1. Pre-Learning

**Figure 6:**
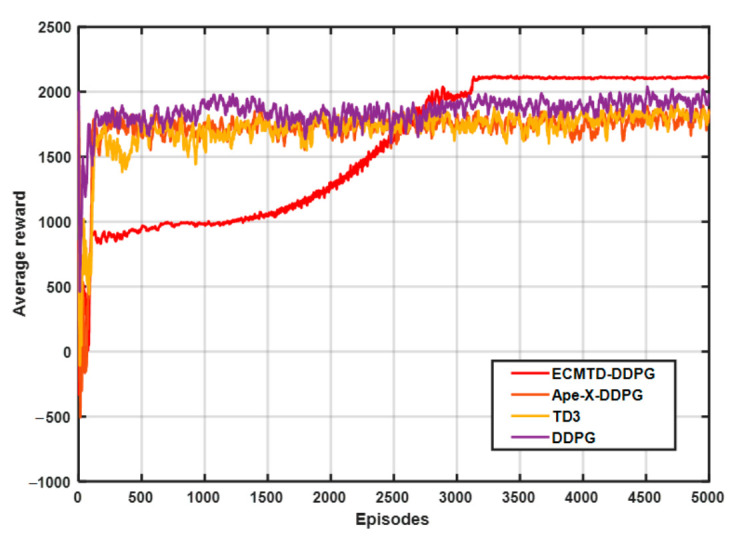
Training diagram.

To ensure the randomness and diversity of samples obtained, the step load current with different amplitudes (0–200 A) is added for training, and the training interval of each episode is 10 s. The training diagram is exhibited in Figure 6.

In Figure 6, the curve represents the average value of rewards in the episode corresponding to algorithms. Among them, the learning speed of Ape-X-DDPG, TD3, and DDPG algorithms is slow, and the learning process has significant fluctuations. In contrast, ECMTD-DDPG has a more stable learning process and a higher final average reward value, indicating that ECMTD-DDPG with edge exploration policy can obtain the optimal solution with a higher value. Meanwhile, due to the distributed exploration method, a large variety of explorers simultaneously explore the optimal solution, making ECMTD-DDPG converge to the optimal solution earlier. This demonstrates that the distributed training method can improve the quality of the trained solution.

### 5.2. Online Application

Two conditions (step load and stochastic load) are used in simulation to verify the effectiveness of the method. Besides, the reinforcement learning (RL) integrated controller (such as Ape-X-DDPG [21], TD3 [20], and DDPG [14] controller), the conventional controller (such as PSO optimized fuzzy-PID (PSO-fuzzy-PID)), fuzzy PID controller (fuzzy-PID), PSO-optimized PID(PSO-PID) controller, and PID controller are introduced as comparisons.

#### 5.2.1. Step Load Condition

**Figure 7:**
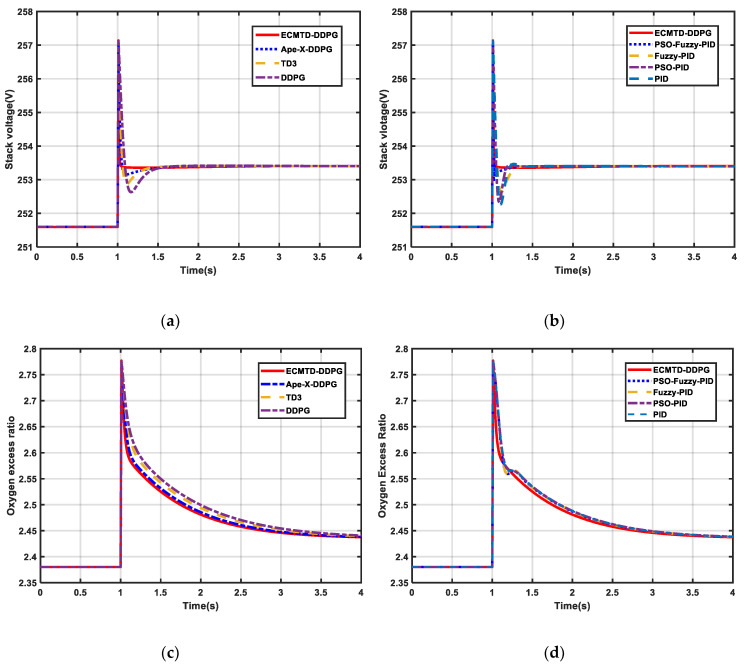

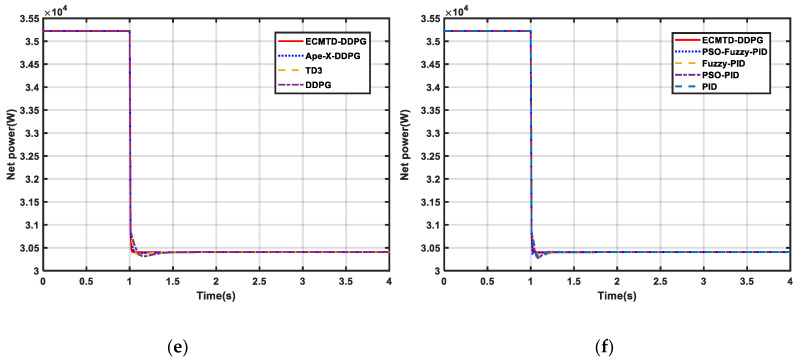
Result of PEMFC under step load condition (**a**) Stack voltage of PEMFC by RL algorithm (**b**) Stack voltage of PEMFC by conventional algorithm (**c**) OER of PEMFC by RL algorithm (**d**) OER of PEMFC by conventional algorithm (**e**) Net power of PEMFC by RL algorithm (**f**) Net power of PEMFC by conventional algorithm.

(1) As presented in Figure 7a–d, the ECMTD-DDPG controller can adapt to a rapid change of OER and output voltage with small overshoot and short dynamic response time compared with all comparative algorithms. Besides, it has fast response, small overshoot, short stabilization time, and does not cause oxygen saturation or starvation in the air flow control. In contrast, the control performance of conventional control algorithms is lower, and the stabilization time of OER is longer.

(2) According to the result of the conventional controller in Table 2, their OER steady time is up to 1.09 times of ECMTD-DDPG, and their output voltage steady time can reach 0.224 s, which is more than 9.6667 times of ECMTD-DDPG. The output voltage overshoot of conventional controllers is all higher than 0.06%, which is more than 20 times that of the ECMTD-DDPG and up to 148.8 times that of ECMTD-DDPG. This is because the serious discordance between controllers leads to the slow response and oscillation in the process of disturbance and easily causes severe instability of the stack voltage.

(3) Furthermore, according to the result of the RL controller in Table 2, the OER steady time of some RL controllers exceeds 4 s, and their output voltage steady time is up to 0.5 s, which is more than 16.7 times of ECMTD-DDPG. Moreover, the output voltage overshoot of other RL controllers are all greater than 0.096%, which is more than 32 times that of the ECMTD-DDPG and up to101 times that of ECMTD-DDPG. Consequently, the ECMTD-DDPG controller has a smaller overshoot and faster response rate compared with other RL controllers. Particularly, the control performance of some RL controllers, such as the DDPG controller, is worse than conventional controllers since the DDPG controller does not have robustness to adapt to every load condition. However, the ECMTD-DDPG controller can have better control performance than all the other controllers.

This is because the exploration policies of Ape-X-DDPG, TD3, and DDPG are too narrow, resulting in convergence to the local optimal solution. Meanwhile, ECMTD-DDPG has been improved to obtain a control policy with better performance. It can be observed by carefully analyzing that the ECMTD-DDPG controller significantly improves the efficiency of algorithm exploration by adopting the distributed edge-cloud collaborative training framework in the training to make full use of all the computing power of GPU and CPU. Additionally, there is no coordination problem between controllers in the control process.

Similarly, it can be observed from Figure 7e–f that the ECMTD-DDPG controller can also ensure the stability of net power in the course of change load. Therefore, the ECMTD-DDPG controller has a better control performance and stability under the condition of step load.

#### 5.2.2. Stochastic Load Condition

**Figure 8:**
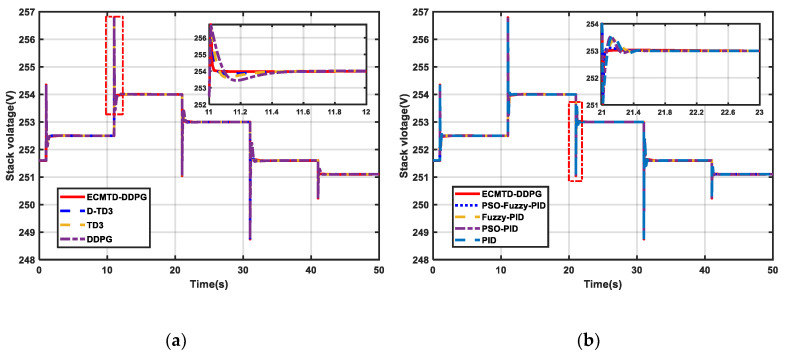

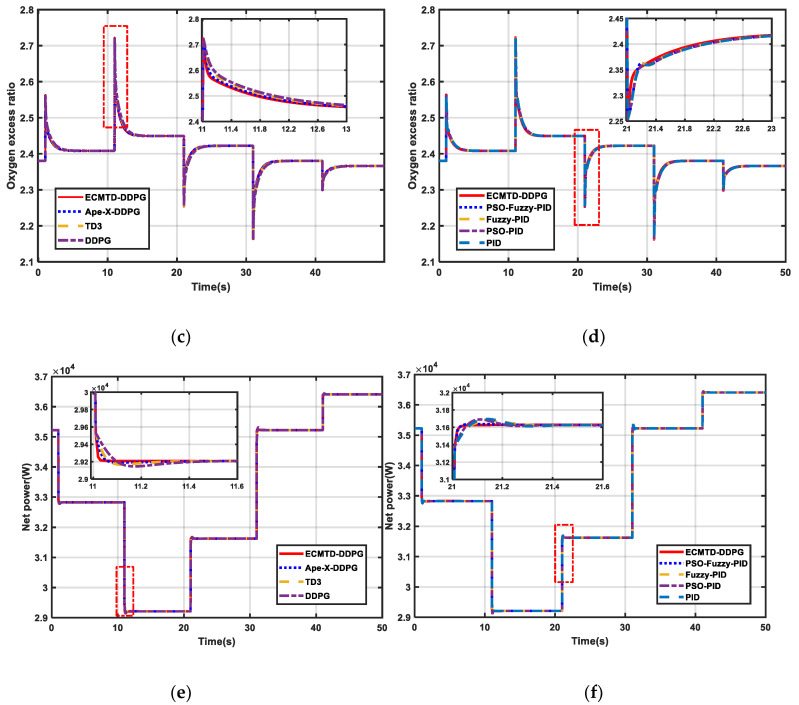
Simulation result of PEMFC under stochastic load (**a**) Stack voltage by RL algorithm (**b**) Stack voltage by conventional algorithm (**c**) OER by RL algorithm (**d**) OER by conventional algorithm (**e**) Net power by RL algorithm (**f**) Net power by conventional algorithm.

**Figure A1:**
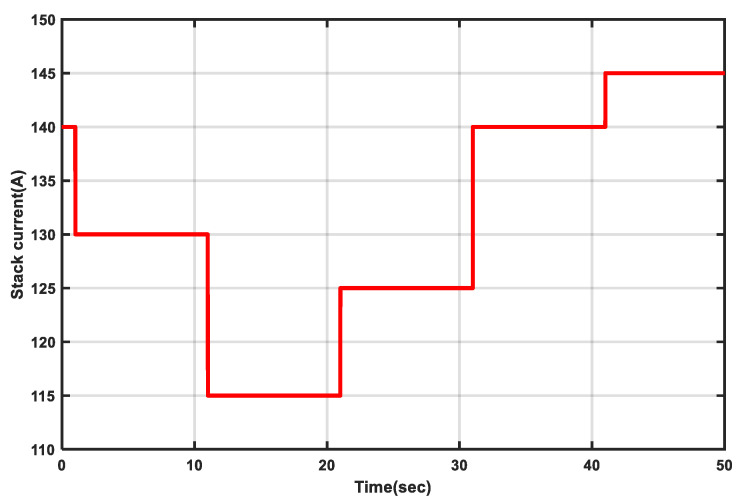
Stack current of PEMFC with Stochastic load condition.

Simulation is performed with stochastic load adding to the system in the simulation to verify the robustness and control performance of the ECMTD-DDPG controller. The simulation time is 50 s. The load current is shown in Figure A1 of Appendix A. The results are illustrated in Figure 8a–f.

As indicated in Figure 8a–d, the ECMTD-DDPG controller has extremely high adaptive ability and robustness. By replacing the conventional dual-controller structure with agent that can realize MIMO control, it can achieve the coordination between different controllers in the gas supply system and automatically make the optimal decision in the current state according to the state of PEMFC, realizing millisecond decision-making of control. Therefore, the proposed algorithm can still output proper OER under stochastic load condition and ensure stable control over the output voltage to achieve better control performance. Particularly, the DDPG controller exhibits completely different control performance under different load conditions because the DDPG controller does not have robustness; this may lead to great overshoot or longer steady time of OER and output voltage.

Under stochastic load condition, the ECMTD-DDPG controller can still realize stable regulation and accurate track of output voltage (Figure 8a–b). Thus, the change in output power is further stabilized (Figure 8e–f).

To sum up, ECMTD-DDPG controller has the characteristics of short response time and rapid response in gas supply control under stochastic load disturbance.

## 6. Conclusions

(1) In this paper, an integrated controller for PEMFC gas supply system control is presented. The original airflow controller and hydrogen flow controller are combined using a deep reinforcement learning algorithm, realizing the coordination between controllers in the gas supply system.

(2) For this framework, the ECMTD-DDPG algorithm is proposed. In this algorithm, the edge exploration policy is adopted, suggesting that the edge explorers including DDPG algorithm, SAC algorithm, and conventional control algorithms are utilized to conduct distributed exploration in the environment, so as to improve the exploration efficiency. Moreover, the classified experience replay mechanism is introduced to improve training efficiency. Besides, clipped multi-Q learning, delay policy updating, and smooth regularization of target policy are combined with the cloud centralized training policy to address the overestimation of Q-value in DDPG. Finally, an adaptive reinforcement learning control algorithm with better global searching ability and training efficiency is obtained, ensuring stable output voltage and OER by realizing MIMO control in PEMFC.

(3) According to the simulation results, it is concluded that ECMTD-DDPG integrated controller can meet the real-time control requirements in PEMFC gas supply control system under different load conditions. It has small overshoot and can effectively avoid sudden change of output voltage, as well as problems of oxygen saturation and starvation. Thus, it can more efficiently control the output voltage of PEMFC.

## Figures and Tables

**Table 1:** Parameter setting.

Parameter	Value
Learning rate of critic	0.001
Learning rate of actor	0.001
Discount factor	0.99
Number of CA-edge-explorers	7
Number of DDPG-edge-explorers	24
variance of *m*th Gaussian-RL-explorer	0.06 + 0.007 × *m*
variance of *j*th OU-RL-explorer	0.03 + 0.005 × *j*
Probability ε of *l*th ε-RL-explorer	0.9
Interval of policy network update	2
Sizes of experience pools 1 and 2	1,000,000
Target action noise variance	0.005

**Table 2:** Response parameters of PEMFC gas supply system.

Type	Parameters	EILMMA-DDPG	APE-X-DDPG	TD3	DDPG	PSO-Fuzzy-PID	Fuzzy-PID	PSO-PID	PID
OER	Rise time T*_r_*/10^−3^ s	1.02	1.09	1.12	1.18	1.29	1.22	1.5	1.60
	Stable time T*_s_*/s	3.52	3.73	>4	>4	3.79	3.80	3.84	3.83
	Overshoot σ/%	14.0068	14.0068	14.0068	14.0069	14.0068	14.0068	14.0069	14.0068
Output voltage	Rise time T*_r_*/10^−3^ s	31	90	110	170	40	110	80	100
	Stable time T*_s_*/s	0.03	0.46	0.39	0.50	0.21	0.29	0.18	0.224
	Overshoot σ/%	0.003	0.096	0.2035	0.3030	0.0669	0.3062	0.4210	0.4464

## Data Availability

Data sharing not applicable. No new data were created or analyzed in this study. Data sharing is not applicable to this article.
